# Association of Sedentary Time with Mortality Independent of Moderate to Vigorous Physical Activity

**DOI:** 10.1371/journal.pone.0037696

**Published:** 2012-06-13

**Authors:** Annemarie Koster, Paolo Caserotti, Kushang V. Patel, Charles E. Matthews, David Berrigan, Dane R. Van Domelen, Robert J. Brychta, Kong Y. Chen, Tamara B. Harris

**Affiliations:** 1 National Institute on Aging, Intramural Research Program, Laboratory of Epidemiology, Demography, and Biometry, Bethesda, Maryland, United States of America; 2 Department of Social Medicine, CAPHRI School for Public Health and Primary Care, Maastricht University, Maastricht, The Netherlands; 3 Institute of Sports Science and Clinical Biomechanics, University of Southern Denmark, Odense, Denmark; 4 Division of Cancer Epidemiology and Genetics Nutritional Epidemiology, National Cancer Institute, Rockville, Maryland, United States of America; 5 Applied Research Program, Division of Cancer Control and Population Sciences, National Cancer Institute, Bethesda, Maryland, United States of America; 6 National Institute of Diabetes and Digestive and Kidney Diseases, Diabetes, Endocrinology, and Obesity Branch, Bethesda, Maryland, United States of America; University of Granada, Spain

## Abstract

**Background:**

Sedentary behavior has emerged as a novel health risk factor independent of moderate to vigorous physical activity (MVPA). Previous studies have shown self-reported sedentary time to be associated with mortality; however, no studies have investigated the effect of objectively measured sedentary time on mortality independent of MVPA. The objective our study was to examine the association between objectively measured sedentary time and all-cause mortality.

**Methods:**

7-day accelerometry data of 1906 participants aged 50 and over from the U.S. nationally representative National Health and Nutrition Examination Survey (NHANES) 2003–2004 were analyzed. All-cause mortality was assessed from the date of examination through December 31, 2006.

**Results:**

Over an average follow-up of 2.8 years, there were 145 deaths reported. In a model adjusted for sociodemographic factors, lifestyle factors, multiple morbidities, mobility limitation, and MVPA, participants in third quartile (hazard ratio (HR):4.05; 95%CI:1.55–10.60) and fourth quartile (HR:5.94; 95%CI: 2.49–14.15) of having higher percent sedentary time had a significantly increased risk of death compared to those in the lowest quartile.

**Conclusions:**

Our study suggests that sedentary behavior is a risk factor for mortality independent of MVPA. Further investigation, including studies with longer follow-up, is needed to address the health consequences of sedentary behavior.

## Introduction

Low physical activity levels are a well-known risk factor of mortality. Previous studies have shown that people who do not meet the physical activity recommendations or those who report less moderate to vigorous activity (MVPA) are at increased risk of death [1], [2], [3], [4]. Sedentary behavior has emerged as a potential risk factor independent of MVPA and is defined as engaging in behaviors during the waking day that are done while sitting or reclining and that result in little energy expenditure above rest, such as using the computer, watching television, driving a car, or sitting at a desk [5].

Recent studies with objectively measured sedentary time data have shown that prolonged time in sedentary behaviors is a cardiometabolic risk factor independent of moderate to vigorous physical activity [6], [7]. Additionally, self-reported sedentary time in several domains including sitting [8], [9], riding in a car [10], and TV watching [11], [12] is positively associated with mortality. These studies are limited by their use of self-reported data, which is known to be prone to reporting errors of physical activity duration and intensity [13]. To our knowledge no studies have investigated the effect of objectively measured sedentary behavior on mortality and whether this association is independent of MVPA. To address this question, we used data from the U.S. nationally representative National Health and Nutrition Examination Survey (NHANES) of adults 50 years of age and older to examine the association between objectively measured sedentary behavior and all-cause mortality.

## Methods

### Ethics Statement

The National Center for Health Statistics Research Ethics Review Board approved the survey protocols, and written informed consent was obtained for all subjects.

### Study Population

This study used data from the 2003–2004 NHANES survey, a nationally representative survey with a stratified, multistage design [14]. For the present study we included 1941 participants aged 50 and older with at least 1 valid day of accelerometry data, defined as ≥10 hours of wear time. We excluded 35 participants with missing data on mortality or covariates, leaving 1906 participants for the present analysis. The NHANES data were linked to death certificate data from the National Death Index and all-cause mortality data were analyzed from the date of examination through December 31, 2006 [15].

**Table 1:** Baseline characteristics of the study population by quartiles of sedentary time.

	**Total**	**Quartiles of Sedentary Time**			
**Characteristic**	**N = 1906**	**1 (lowest)** **N = 476**	**2** **N = 477**	**3** **N = 477**	**4 (highest) N = 476**
Age (years), mean (SD)*	63.8 (10.5)	60.1(8.9)	62.4(9.7)	64.6(10.5)	68.6(11.2)
Women, n(%)	54.0	53.1	53.6	54.1	55.2
Ethnicity, %					
Non-Hispanic white	79.5	74.1	80.6	84.5	78.3
Non-Hispanic black	9.2	11.7	8.4	5.2	12.5
Hispanic	3.8	7.2	3.0	2.3	2.9
Other	7.4	7.1	8.0	8.1	6.3
Education, %					
<High school	21.0	23.8	20.1	18.7	21.9
High school	26.3	28.7	24.2	26.1	26.5
>High school	52.7	47.5	55.7	55.3	51.6
Smoking, %					
Never	45.3	43.6	41.8	50.1	45.8
Former	38.3	39.3	38.2	36.0	40.2
Current	16.4	17.1	20.0	13.9	14.0
Alcohol, %					
Never	14.5	12.3	13.5	15.6	17.0
Former	26.5	24.6	26.0	27.3	28.4
Current	54.7	57.6	57.5	53.3	49.6
Missing	4.2	5.5	3.0	3.7	5.0
BMI (kg/m^2^), mean (SD)	28.5(5.7)	28.7(5.5)	27.9(5.4)	28.3(5.6)	29.1(6.4)
Diabetes, %	15.0	11.1	11.2	15.8	22.7
Coronary heart disease, %	8.5	6.5	7.7	9.9	9.9
Congestive heart failure, %	5.6	2.6	4.8	6.9	8.4
Stroke,%	5.4	4.7	5.1	4.6	7.5
Cancer, %	18.1	12.8	17.1	21.2	21.7
Mobility limitation, %	18.3	16.1	17.1	17.6	22.9
Sedentary time per day(hours), mean (SD)	9.0 (2.3)	6.3(1.0)	8.2(0.5)	9.6(0.5)	12.2(2.0)
% sedentary time, mean (SD)	62.1(12.2)	48.3(8.2)	59.0(6.6)	66.6(6.7)	75.6(8.0)
Minutes of moderate to vigorous activity per day, mean (SD)	14.2.1(17.4)	20.8(21.2)	14.3(16.1)	13.7(17.1)	7.3(10.7)

**Figure 1:**
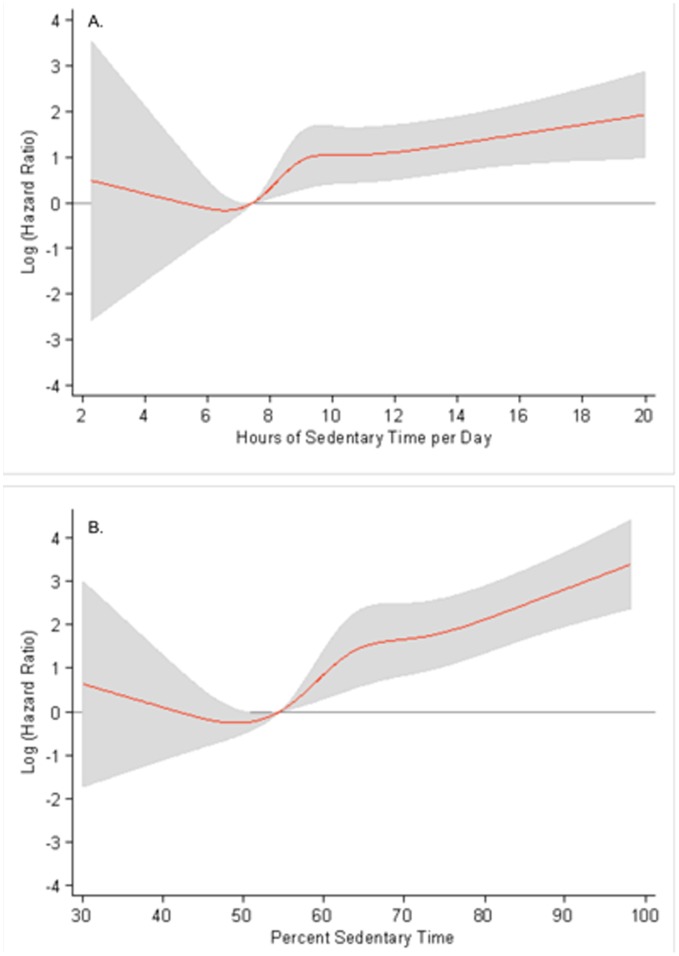
Log hazard ratio (with 95%CI bands), for hours of sedentary time (A) and percent sedentary time (B). ^a^ Adjusted for age, gender, race/ethnicity, and educational level with the 25th percentile as the reference points.

**Figure 2:**
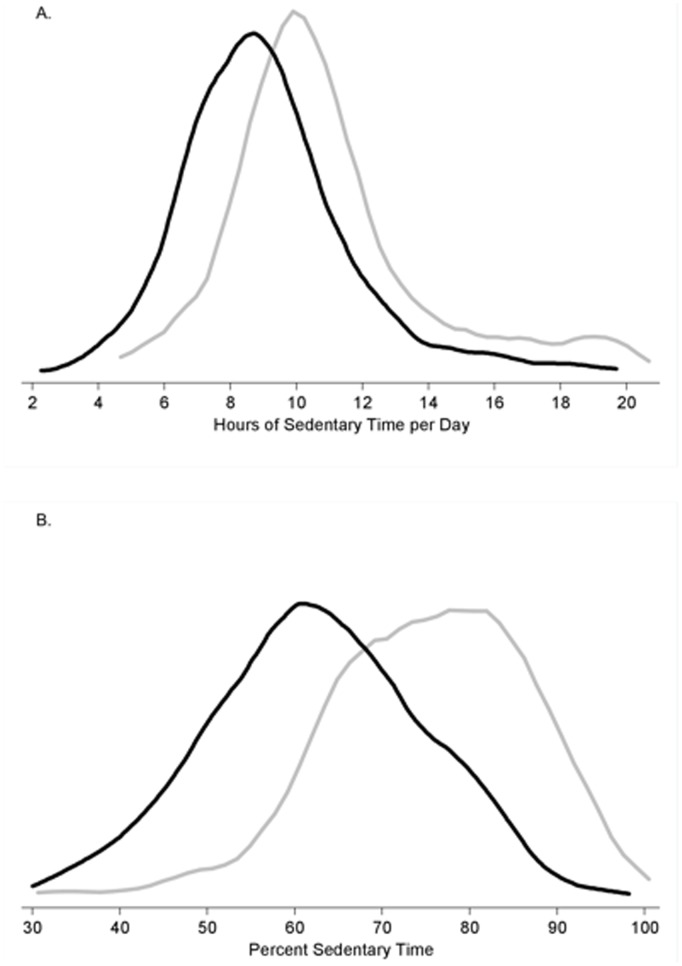
Distribution of hours of sedentary time per day (A) and percent sedentary time (B) by mortality status (black line = survived; gray line = died).

#### Measures

*Accelerometry.* Physical activity was assessed using the uniaxial ActiGraph AM-7164 accelerometer (ActiGraph, Ft. Walton Beach, FL). Participants were instructed to wear the monitor while awake for seven consecutive days on a belt around their waist [16]. Non-wear time was defined as intervals of at least 60 consecutive minutes of zero counts, with allowance for up to two minutes of counts between 1 and 100. Sedentary time in hours was defined as counts per minute below 100 during detected wear time [17]. Percent sedentary time was calculated as total sedentary time divided by total wear time. Minutes of MVPA was defined as counts per minute ≥2020 [16].

**Table 2:** Mortality rates and hazard ratios and 95% confidence intervals of mortality according to quartiles of sedentary time.

			Model 1^c^		Model 2^d^		Model 3^e^	
	N	Mortality rate^b^	HR	95%CI	HR	95%CI	HR	95%CI
Hours of sedentary time per day^a^								
1 (lowest)	476	8	1.00 (REF)		1.00 (REF)		1.00 (REF)	
2	477	18	2.19	1.07–4.51	1.98	0.95–4.13	1.74	0.81–3.73
3	477	32	3.81	1.62–.9.00	3.31	1.56–7.03	2.74	1.35–5.52
4 (highest)	476	53	4.93	2.08–11.68	4.13	1.89–9.05	3.26	1.59–6.69
Percent sedentary time^a^								
1 (lowest)	475	5	1.00 (REF)		1.00 (REF)		1.00 (REF)	
2	481	10	2.10	0.93–4.76	1.86	0.82–4.22	1.53	0.60–3.89
3	482	30	7.08	3.29–15.23	5.73	2.36–13.88	4.05	1.55–10.60
4 (highest)	468	69	11.93	4.96–28.71	9.02	3.76.21.63	5.94	2.49–14.15

a Quartile cut-off point sedentary time men: 7.6, 9.2, 10.8 hrs, women: 7.2, 8.7 10.1 hrs; percent sedentary time men: 55.4, 64.7, 73.5%, women: 53.9, 61.9, 70.5%.

b Mortality rate per 1000 person years.

c Model 1: Adjusted for gender, age, race/ethnicity, educational level.

d Model 2: Adjusted for gender, age, race/ethnicity, educational level, smoking status, alcohol intake, BMI, diabetes, coronary heart disease, congestive heart failure, cancer, stroke, and mobility limitation.

e Model 3: Adjusted for gender, age, race/ethnicity, educational level, smoking status, alcohol intake, BMI, diabetes, coronary heart disease, congestive heart failure, cancer, stroke, mobility limitation, and moderate to vigorous physical activity.

#### Covariates

Covariates included age, gender, race/ethnicity (non-Hispanic white, non-Hispanic black, Hispanic, other), education (less than high school, high school or general equivalency diploma, more than high school), alcohol consumption (never, former, current drinker, missing), smoking status (never, former, current), body mass index (BMI, kg/m^2^), self-reported diabetes, coronary heart disease, congestive heart failure, stroke, cancer, and self-reported mobility limitation defined as any difficulty walking a quarter of a mile or walking up ten steps without resting.

**Table 3:** Hazard ratios and 95% confidence intervals of mortality according to quartiles of sedentary time in different subsamples^a^.

	Model 1^c^		Model 2^d^		Model 3^e^		Model 4^f^	
	HR	95%CI	HR	95%CI	HR	95%CI	HR	95%CI
Hours of sedentary time^b^								
1 (lowest)	1.00 (REF)		1.00 (REF)		1.00 (REF)		1.00 (REF)	
2	0.93	0.40–2.14	3.06	1.26–4.75	1.97	0.66–5.91	1.92	0.50–7.32
3	2.02	0.78–5.23	1.59	0.59–4.26	5.01	1.39–18.08	3.02	1.21–7.55
4 (highest)	2.62	1.15–5.99	4.16	1.22–14.16	8.31	2.54–27.18	4.62	1.76–12.11
Percent sedentary time^b^								
1 (lowest)	1.00 (REF)		1.00 (REF)		1.00 (REF)		1.00 (REF)	
2	0.89	0.28–2.89	0.95	0.26–3.50	1.85	1.10–7.79	1.96	0.55–7.04
3	2.57	0.98–6.72	2.53	0.96–4.70	1.97	0.87–4.43	4.78	1.38–16.65
4 (highest)	4.24	1.76–10.24	3.34	1.10–10.18	2.92	1.10–7.80	6.49	2.05–20.61

a Adjusted for gender, age, race/ethnicity, educational level, smoking status, alcohol intake, BMI, diabetes, coronary heart disease, congestive heart failure, cancer, stroke, mobility limitation, and moderate to vigorous physical activity.

b Quartile cut-off point sedentary time men: 7.6, 9.2, 10.8 hrs, women: 7.2, 8.7 10.1 hrs; percent sedentary time: men: 55.4, 64.7, 73.5%, women: 53.9, 61.9, 70.5%.

c Excluded participants with mobility limitation, n = 1518, 111 deaths.

d Excluded participants with diabetes, coronary heart disease, congestive heart failure, and stroke n = 1326, 70 deaths.

e Excluded participants with cancer, n = 1583, 96 deaths.

f Excluded first year of follow-up, n = 1855, 94 deaths.

#### Statistical analysis

Cox proportional hazard models were fitted to estimate the hazard ratios (HR) of gender-specific quartiles of each sedentary variable on time to death. Interactions with gender and age were not statistically significant (all p>0.10); therefore all results are shown for men and women and all ages combined. Data were analyzed using SAS Software 9.1 (SAS Institute Inc., Cary, NC) and SUDAAN 10.0 (RTI International, Research Triangle Park, NC) to account for the complex survey design and incorporate sample weights. Restricted cubic splines (Stata SE 10.1) were used to graphically illustrate the relation between time spent in sedentary behavior and mortality, the lowest quartile cutpoint was used as a reference point.

**Table 4:** Mortality rates and hazard ratios and 95% confidence intervals of mortality according to quartiles of sedentary time selecting participants with 4 or more valid days of accelerometry data (n = 1711).

	N	Mortality rate^b^	HR c	95%CI
Hours of sedentary time per day^a^				
1 (lowest)	427	7	1.00 (REF)	
2	428	14	1.16	0.48–2.83
3	428	32	2.94	1.42–6.08
4 (highest)	428	50	3.22	1.39–7.44
Percent sedentary time^a^				
1 (lowest)	427	4	1.00 (REF)	
2	432	10	2.27	0.65–7.97
3	431	28	5.25	1.30–21.23
4 (highest)	421	63	7.26	2.33–22.60

a Quartile cut-off point sedentary time men: 7.6, 9.2, 10.8 hrs, women: 7.2, 8.7 10.1 hrs; percent sedentary time: men: 55.4, 64.7, 73.5%, women: 53.9, 61.9, 70.5%.

b Mortality rate per 1000 person years.

c Adjusted for gender, age, race/ethnicity, educational level, smoking status, alcohol intake, BMI, diabetes, coronary heart disease, congestive heart failure, cancer, stroke, mobility limitation and moderate to vigorous physical activity.

**Table 5:** Mortality rates and hazard ratios and 95% confidence intervals of mortality according to quartiles of moderate to vigorous physical activity.

	N	Mortalityrate^b^	HR^c^	95%CI
Minutes of moderate to vigorousphysical activity time per day				
1 (highest)	476	6	1.00 (REF)	
2	479	8	1.10	0.38–3.21
3	469	32	3.87	1.44–10.40
4 (lowest)	482	68	5.60	2.25–13.96

b Mortality rate per 1000 person years.

c Adjusted for gender, age, race/ethnicity, educational level, smoking status, alcohol intake, BMI, diabetes, coronary heart disease, congestive heart failure, cancer, stroke, and mobility limitation.

## Results

Baseline characteristics of the study population according to quartiles of sedentary time are shown in Table 1. Over an average follow-up of 2.8 years, 145 deaths were reported. Figure 1 shows that mortality risk increased significantly with greater sedentary time adjusted for sociodemographic factors. Figure 2 clearly illustrates the shifted distribution of more time spent sedentary in those who died compared with those who survived.

Table 2 shows mortality rates per 1000 person years and hazard ratios of mortality according to quartiles of sedentary time. In model 3 that included sociodemographic factors, lifestyle factors, multiple morbidities, mobility limitation, and MVPA, those in the highest quartile of sedentary hours had a 3.3 times increased risk of mortality compared to participants in lowest quartile (95% confidence interval (CI):1.59–6.69). The percent sedentary time was also strongly associated with mortality. Participants in the third quartile (hazard ratio (HR):4.05; 95%CI:1.55–10.60) and fourth quartile (HR:5.94; 95%CI:2.49–14.15) of percent sedentary time had a significantly increased risk of death compared to those in the lowest quartile.

In sensitivity analysis that evaluated the potential for these associations to be explained by reverse causality, we excluded participants with mobility limitation (Table 3, Model 1), diabetes, disease coronary heart disease, congestive heart failure, and stroke (Model 2), and cancer (Model 3). In these groups of healthier individuals, sedentary time remained positively associated with mortality. Further, after excluding the first year of follow-up to exclude deaths (n = 55) in the first year of the study the positive association between sedentary time and mortality remained (Table 3, Model 4). When we selected participants with 4 or more valid days (n = 1701) of accelerometry data instead of the 1 or more valid day in the current analysis, results remained very similar (Table 4).

Table 5 shows mortality rates per 1000 person years and hazard ratios of mortality according to quartiles of MVPA time. Those in the lowest quartile of MVPA had 5.6 times greater mortality risk compared to those in the highest quartile of MVPA (95%CI: 2.25–13.96) in a model that was adjusted for sociodemographic factors, lifestyle factors, multiple morbidities, and mobility limitation.

## Discussion

This study shows that time spent in sedentary behavior is positively associated with mortality in this representative sample of adults aged 50 and older. Participants in the highest quartile of percentage of time spent sedentary, which corresponds to more than 73.5% of time in men and more than 70.5% in women, had more than 5 times greater risk of death compared to those in the lowest quartile. Importantly, these associations were independent of MVPA. MVPA was also strongly associated with mortaliy. Recent studies have identified sedentary behavior as a novel risk factor of cardiometabolic disease [6], [7], [18]. To our knowledge the present study is the first to show that objectively measured sedentary time also is an important risk factor of mortality, even after adjusting for MVPA.

Overall, despite growing evidence from epidemiological studies indicate that sedentary behavior is a health risk factor relatively little is still known about the physiological responses caused by sedentariness (inactivity physiology). Sedentary time has been associated with adverse alterations in metabolic risk factors (e.g. triglycerides, fasting glucose, and high density lipoprotein (HDL) cholesterol) [6], [7] which may explain the higher mortality risk. Animal studies have shown that sedentary time substantially suppresses skeletal muscle lipoprotein lipase (LPL) (a protein important for controlling plasma triglyceride catabolism, HDL cholesterol, and other metabolic risk factors) [19]. Furthermore, sedentary behavior has been recently associated with mitochondrial dysfunction, dysregulation of cellular redox status and increased inflammation in older sedentary adults compared to more active adults [20]. Sedentary behavior in addition to aging seems thereby associated with reduced mitochondrial biogenesis and increased electron leak from the mitochondrial electron transport chain which exposes the skeletal muscle intracellular milieu to increased reactive oxygen species – mediated toxicity and altered mitochondrial DNA deletions and mutations. Ultimately, this chain of events leads to accelerated cellular senescence and cell death [20]. Nevertheless, more research is warranted into the mechanisms underlying the adverse health effects of sedentariness.

An important strength of our study was that sedentary behavior was measured objectively with accelerometry. However, accelerometers are not sensitive to detect all activities such as standing still, upper body movements and biking. Use of an accelerometer definition of MVPA based on studies of primarily younger adults may lead to an underestimate of MVPA for older adults due to the decline in exercise capacity with age, which may lead to residual confounding. Similarly, while the cutpoint we used to define sedentary time has been evaluated for validity [17], it is possible that the accuracy of this threshold varies in different age groups. However, small variations in the cut-point selected (e.g., 50, 100, 150 counts per minute) would not be likely to affect the classification of individuals into quartiles of sedentary time. Thus, our results are unlikely to be affected by the cutpoint chosen to define sedentary time. We adjusted our analyses for a wide range of potential confounding variables, we can however not exclude that other unknown or unmeasured factors may account for some of the reported associations. The follow-up time of our study was short which may increase the risk of reverse causation. However, to address this issue, in separate models we excluded individuals with mobility limitation, pre-existing disease, and early deaths and the positive association between sedentary time and mortality remained. Further studies with longer mortality follow-up are needed to corroborate our findings. Larger studies will also enable us to evaluate the joint or synergistic effects of sedentary time and time spent in physical activities of different intensities on adverse health outcomes. Also, future studies examining the effect of different cutpoints to control for physical activity would be instructive.

In conclusion, our study supports the importance of sedentary behavior as a novel risk factor of mortality independent of MVPA. Considering our results and the results of other studies showing the predominance of sedentary behavior in daily life and the importance of prolonged time spend in these sedentary behaviors as a health risk factor, it is essential to both promote MVPA participation and also support reduction of prolonged periods of sedentary time. A recent Position Stand from the American College of Sports Medicine on the quantity and quality of exercise in adults mentions the importance to reduce sedentary time irrespective of a person’s exercise habit [21]. The current physical activity guidelines primarily focus on MVPA [1]; a recommendation to reduce sedentary time might be an important consideration for discussion of new or updated physical activity guidelines.
